# Supplementary material for: Investigating the epidemiological relevance of secretory otitis media and neighboring organ diseases through an Internet search
Source: PeerJ. 2024 Mar 5;12:e16981. doi: 10.7717/peerj.16981 (PMC10921933; doi:10.7717/peerj.16981)
Supplement: Table S1 — The variable from Baidu index: average daily search volume (D) of every term. 2) Monthly search volume (M) is calculated as the average daily search volume for the month multiplied by the number of days in that month (n). (e.g., The average daily search volume of February 2011 is 576 times, then the monthly search volume in February 2011 equals 16128 times (576*28 = 16128). 3) Annual search volume (Y) is then obtained by summing up the monthly search volumes for the entire year. Results are presented as percentages: 4) The percentages of monthly search volume (P) are calculated by dividing the monthly search volume for the year by the annual search volume. 5) The percentages of average monthly search volume (Pm) are obtained by summing the monthly search volumes for the same month throughout the year and dividing the result by the total annual search volume. [file peerj-12-16981-s002.docx]

Supplementary Table S1. Variables and calculations

| Variable | Symbol | Formula of calculation | Example (SOM) |
| --- | --- | --- | --- |
| average daily search volume | *D* | Original data from the Baidu Index | *D* (February 2021) = 576 |
| the number of days in the month | *n* | - | *n* (February) = 28 |
| Monthly search volume | *M* | *M = D*n* | *M* (February 2021) = 576*28 =16128 |
| Annual search volume | *Y* | $Y\left( \mathrm{year} \right)=\sum_{i=\mathrm{January}}^{\mathrm{December}} M(i,\mathrm{year})$ | *Y* (2021) = *M* (January 2021) + *M* (February 2021) +…+ *M* (December 2021) = 227754 |
| The percentages of monthly search volume | *P* | $P\left( month year \right)=\frac{M(month year)}{Y(\mathrm{year})}$ | *P* (February 2021) = *M* (February 2021) / *Y* (2021) = 16128/227754 = 0.0708 |
| The percentages of average monthly search volume | *P_m_* | $P_{m}=\frac{\sum_{i=2011}^{2021} M(\mathrm{February} i)}{\sum_{i=2011}^{2021} Y(i)}$ | *P_m_* (February) = [*M* (February 2011) + *M* (February 2012) +…+ *M* (February 2021)] / [*Y* (2011) + *Y* (2012) +…+ *Y* (2021)] = 0.0722 |

1. The variable from Baidu index: **average daily search volume (*D*)** of every term.
2. **Monthly search volume (*M*)** is calculated as the average daily search volume for the month multiplied by the number of days in that month (*n*). (e.g. The average daily search volume of February 2011 is 576 times, then the monthly search volume in February 2011 equals 16128 times (576*28 = 16128).
3. **Annual search volume (*Y*)** is then obtained by summing up the monthly search volumes for the entire year.

Results are presented as percentages:

1. **The percentages of monthly search volume (*P*)** are calculated by dividing the monthly search volume for the year by the annual search volume.
2. **The percentages of average monthly search volume** **(*P_m_*)** are obtained by summing the monthly search volumes for the same month throughout the year and dividing the result by the total annual search volume.
