# Supplementary material for: Investigating the epidemiological relevance of secretory otitis media and neighboring organ diseases through an Internet search
Source: PeerJ. 2024 Mar 5;12:e16981. doi: 10.7717/peerj.16981 (PMC10921933; doi:10.7717/peerj.16981)
Supplement: Table S2 [file peerj-12-16981-s003.docx]

Supplementary Table S2. Average monthly search volume

| MONTH | SOM | AR | Rhinosinusitis | NSD | NPC | AH | Tonsillitis | Pharyngolaryngitis | GERD |
| --- | --- | --- | --- | --- | --- | --- | --- | --- | --- |
| Jan | 0.08 | 0.05 | 0.09 | 0.08 | 0.08 | 0.08 | 0.08 | 0.08 | 0.08 |
| Feb | 0.07 | 0.05 | 0.07 | 0.07 | 0.07 | 0.07 | 0.07 | 0.07 | 0.07 |
| Mar | 0.09 | 0.08 | 0.09 | 0.09 | 0.09 | 0.08 | 0.09 | 0.09 | 0.09 |
| Apr | 0.09 | 0.09 | 0.09 | 0.09 | 0.08 | 0.08 | 0.08 | 0.09 | 0.08 |
| May | 0.09 | 0.10 | 0.09 | 0.09 | 0.11 | 0.09 | 0.09 | 0.09 | 0.08 |
| Jun | 0.08 | 0.08 | 0.08 | 0.08 | 0.08 | 0.08 | 0.09 | 0.08 | 0.09 |
| Jul | 0.08 | 0.08 | 0.07 | 0.09 | 0.08 | 0.10 | 0.09 | 0.08 | 0.09 |
| Aug | 0.08 | 0.13 | 0.07 | 0.09 | 0.08 | 0.09 | 0.08 | 0.09 | 0.09 |
| Sep | 0.08 | 0.12 | 0.07 | 0.08 | 0.08 | 0.06 | 0.08 | 0.08 | 0.09 |
| Oct | 0.08 | 0.09 | 0.08 | 0.08 | 0.08 | 0.08 | 0.08 | 0.08 | 0.08 |
| Nov | 0.08 | 0.07 | 0.09 | 0.08 | 0.08 | 0.09 | 0.08 | 0.08 | 0.08 |
| Dec | 0.09 | 0.06 | 0.10 | 0.09 | 0.08 | 0.10 | 0.09 | 0.09 | 0.08 |
