# Supplementary material for: Investigating the epidemiological relevance of secretory otitis media and neighboring organ diseases through an Internet search
Source: PeerJ. 2024 Mar 5;12:e16981. doi: 10.7717/peerj.16981 (PMC10921933; doi:10.7717/peerj.16981)
Supplement: Table S3 [file peerj-12-16981-s004.docx]

Supplementary Table S3. The search volume during the first 5 months from 2019 to 2021

|  | Year | Jan | Feb | Mar | Apr | May |
| --- | --- | --- | --- | --- | --- | --- |
| SOM | 2019 | 27900 | 22260 | 28179 | 28770 | 29171 |
|  | 2020 | 21390 | 14993 | 18042 | 18600 | 17484 |
|  | 2021 | 18414 | 16128 | 20212 | 20250 | 20894 |
| AR | 2019 | 81375 | 69384 | 133486 | 170820 | 168392 |
|  | 2020 | 83452 | 103269 | 95976 | 89850 | 78182 |
|  | 2021 | 60357 | 57120 | 100843 | 117330 | 105617 |
| Rhinosinusitis | 2019 | 197439 | 155008 | 210211 | 218520 | 215884 |
|  | 2020 | 106702 | 92162 | 95542 | 84900 | 78740 |
|  | 2021 | 134540 | 96180 | 119970 | 138180 | 118916 |
| NSD | 2019 | 40672 | 36344 | 49197 | 51510 | 46128 |
|  | 2020 | 35123 | 30885 | 36177 | 35130 | 33976 |
|  | 2021 | 34007 | 29652 | 34968 | 34050 | 33139 |
| NPC | 2019 | 80259 | 61516 | 96131 | 94800 | 72292 |
|  | 2020 | 44702 | 37439 | 51088 | 48210 | 39959 |
|  | 2021 | 55490 | 44744 | 56947 | 52920 | 46190 |
| AH | 2019 | 86428 | 67312 | 82894 | 86580 | 81313 |
|  | 2020 | 61504 | 30508 | 45074 | 52740 | 51863 |
|  | 2021 | 61225 | 51884 | 65193 | 67620 | 89683 |
| Tonsillitis | 2019 | 75578 | 61404 | 81902 | 85410 | 94519 |
|  | 2020 | 91543 | 97237 | 78151 | 64560 | 67797 |
|  | 2021 | 92938 | 65912 | 79918 | 87150 | 87885 |
| Pharyngolaryngitis | 2019 | 521699 | 425712 | 556636 | 503520 | 507594 |
|  | 2020 | 331390 | 392370 | 312108 | 254010 | 240715 |
|  | 2021 | 314340 | 230888 | 295988 | 329640 | 321346 |
| GERD | 2019 | 10354 | 9268 | 11067 | 9900 | 10602 |
|  | 2020 | 8215 | 9918 | 11563 | 9480 | 9331 |
|  | 2021 | 8494 | 7000 | 9207 | 8370 | 7998 |
